# Wogonin alleviates liver injury in sepsis through Nrf2‐mediated NF‐κB signalling suppression

**DOI:** 10.1111/jcmm.16604

**Published:** 2021-05-12

**Authors:** Ji‐Min Dai, Wei‐Nan Guo, Yi‐Zhou Tan, Kun‐Wei Niu, Jia‐Jia Zhang, Cheng‐Li Liu, Xiang‐Min Yang, Kai‐Shan Tao, Zhi‐Nan Chen, Jing‐Yao Dai

**Affiliations:** ^1^ Department of Hepatobiliary Surgery Xijing Hospital Fourth Military Medical University Xi’an China; ^2^ Department of Cell Biology National Translational Science Center for Molecular Medicine Fourth Military Medical University Xi’an China; ^3^ Department of Dermatology Xijing Hospital Fourth Military Medical University Xi’an China; ^4^ Department of Periodontology School of Stomatology Fourth Military Medical University Xi’an China; ^5^ Department of Hepatobiliary Surgery Air Force Medical Center Beijing China; ^6^ Fourth Military Medical University Xi’an China

**Keywords:** liver injury, Nrf2, oxidative stress, sepsis, wogonin

## Abstract

Sepsis is a life‐threatening organ dysfunction syndrome, and liver is a susceptible target organ in sepsis, because the activation of inflammatory pathways contributes to septic liver injury. Oxidative stress has been documented to participate in septic liver injury, because it not only directly induces oxidative genotoxicity, but also exacerbates inflammatory pathways to potentiate damage of liver. Therefore, to ameliorate oxidative stress is promising for protecting liver in sepsis. Wogonin is the compound extracted from the medicinal plant *Scutellaria baicalensis* Geogi and was found to exert therapeutic effects in multiple inflammatory diseases via alleviation of oxidative stress. However, whether wogonin is able to mitigate septic liver injury remains unknown. Herein, we firstly proved that wogonin treatment could improve survival of mice with lipopolysaccharide (LPS)‐ or caecal ligation and puncture (CLP)‐induced sepsis, together with restoration of reduced body temperature and respiratory rate, and suppression of several pro‐inflammatory cytokines in circulation. Then, we found that wogonin effectively alleviated liver injury via potentiation of the anti‐oxidative capacity. To be specific, wogonin activated Nrf2 thereby promoting expressions of anti‐oxidative enzymes including NQO‐1, GST, HO‐1, SOD1 and SOD2 in hepatocytes. Moreover, wogonin‐induced Nrf2 activation could suppress NF‐κB‐regulated up‐regulation of pro‐inflammatory cytokines. Ultimately, we provided in vivo evidence that wogonin activated Nrf2 signalling, potentiated anti‐oxidative enzymes and inhibited NF‐κB‐regulated pro‐inflammatory signalling. Taken together, this study demonstrates that wogonin can be the potential therapeutic agent for alleviating liver injury in sepsis by simultaneously ameliorating oxidative stress and inflammatory response through the activation of Nrf2.

## 1 INTRODUCTION

Sepsis is a life‐threatening syndrome characterized by the dysfunction of multiple organs due to dysregulated host immune response to infection. The incidence of sepsis is about 0.3%‐1.03% in high‐income countries, and its mortality is between 30% and 50% as the leading cause of death in the intensive care units (ICUs).^1^, ^2^, ^3^, ^4^ Of note, septic liver injury substantially contributes to the poor prognosis of patients and is regarded as an independent indicator of the mortality. Recent studies have demonstrated that damaged hepatocytes would release damage‐associated molecular patterns (DAMPs) to trigger more pronounced regional and systemic inflammatory responses, exacerbating the dysfunction of liver and other organs.^5^, ^6^ In addition, septic liver injury is closely associated with cholestasis, hypoxic hepatitis and severe coagulopathy, which would progress to liver failure and worsen the outcome of patients with sepsis. Therefore, to effectively ameliorate liver injury is crucial for the treatment of sepsis and the improvement of patients’ prognosis.

During the progression of sepsis, hypotension‐induced ischaemia, severe infection and dysregulated immune response are all greatly implicated in inducing septic liver injury. The generation of reactive oxygen species (ROS) is tightly linked to these crucial pathogenic insults and mediates their effects. On one hand, oxidative stress with excessive accumulation of ROS can directly cause damage to hepatocytes by promoting the formation of oxidative protein adducts and lipid peroxides.^7^ On the other hand, continuous oxidative stress is a potent trigger of inflammatory signalling to amplify the release of cytokines like tumour necrosis factor‐α (TNF‐α) and interleukin‐6 (IL‐6) via the activation of nuclear factor‐κB (NF‐κB) pathway in hepatocytes and Kupffer cells, thus forming a positive feedback loop to recruit more neutrophils and lymphocytes and augment the damage of liver. Recently, some studies have revealed that the agents with anti‐oxidative capacity could effectively suppress the systemic immune response and improve the outcome of patients with sepsis.^8^, ^9^ However, whether septic liver injury could be relieved by the scavenging of ROS with some specific anti‐oxidative agents and thus bring benefits to sepsis treatment remains far from understood.

Wogonin (5,7‐dihydroxy‐8‐methoxyflavone) is the natural component isolated from the root of *Scutellaria baicalensis* Georgi, the crude drug commonly used for allergies and cancer as complementary treatment.^10^, ^11^ Intriguingly, its anti‐oxidative effect has been extensively proved in multiple diseases, in particular those with the involvement of inflammatory responses. To be specific, wogonin was capable of attenuating oxidative stress via the activation of peroxisome proliferator‐activated receptor‐γ (PPAR‐γ)/adiponectin receptor 2 (AdipoR2) pathway and exerted the therapeutic effect on non‐alcoholic steatohepatitis (NASH) in mice.^12^ Moreover, in drug‐ or heavy metal‐induced nephritis, wogonin exerted its anti‐oxidative capacity through PPAR‐γ and NF‐κB signalling so as to alleviate nephrotoxicity.^13^, ^14^ What's more, wogonin played a neuroprotective role by restraining oxidative stress‐induced chronic inflammation in primary cultured cortical neurons.^15^ Nevertheless, it still remains to be elicited whether wogonin can suppress septic liver injury via the prevention of oxidative stress.

Nuclear erythroid 2‐related factor (Nrf2) is a crucial regulator of redox balance with its transcriptional activity in various tissues and cells. After the translocation to nucleus, Nrf2 binds to the antioxidant response elements (AREs) in DNA of downstream genes and initiates expression of antioxidant enzymes such as haeme oxygenase‐1 (HO‐1) and superoxide dismutases (SODs).^16^ In vivo studies demonstrate that knockout of Kelch‐like ECH‐associated protein 1 (Keap1) can greatly improve the treatment outcome of septic mice via the potentiation of the transcriptional activity and anti‐oxidative capacity of Nrf2.^17^ Besides, in hyperlipidaemia‐ and hyperglycaemia‐induced liver injury model, hydrogen sulphide alleviates liver injury via S‐sulfhydrated‐Keap1/Nrf2 signalling, whereas Nrf2 deficiency can abolish the protective effects, indicating the indispensable role of Nrf2 signalling for maintaining liver homeostasis and preventing liver injury. Recently, the activation of Nrf2 signalling has been proved to be responsible for the protective role of wogonin in different tissues and cells; thus, we suggest that wogonin exerts protective effects towards liver injury via activation of Nrf2 signalling in sepsis.

## 2 MATERIALS AND METHODS

### 2.1 Sepsis mouse model

C57BL/6J mice (male, 7‐8 weeks old weighing 20 ± 2 g) were purchased from Experimental Animal Center of Fourth Military Medical University. The mice were kept under well‐controlled environmental conditions with constant temperature (25 ± 2°C), humidity (60 ± 10%) and alternating 12 hour light‐dark cycles. Standard pellet diet and sterilized water were accessible ad libitum. The investigation was performed in accordance with ethical regulations including International Guiding Principles for Biomedical Research Involving Animals and was approved by the Ethical Committee for Animal Experimentation of Fourth Military Medical University. The research protocol was designed and executed according to the principles of the Declaration of Helsinki.

Wogonin purchased from MCE (MedChemExpress) was dissolved with DMSO (Sigma‐Aldrich) for storage and was then mixed with 40% PEG300 (MedChemExpress), 5% Tween‐80 and 45% sterilized saline to indicated concentrations for intraperitoneal injection (i.p.). The vehicle was used for injection as control. To establish LPS‐induced sepsis mouse model, LPS (Sigma‐Aldrich) was dissolved and diluted with the identical vehicle of wogonin to 8 mg/mL (40 mg/kg) for intraperitoneal injection. Wogonin i.p. was performed 1 hour before LPS injection. Body temperature and respiratory rate were monitored 1 hour before and 8 hours after LPS injection. Body temperature was measured by the Animal Digital Electronic Body Thermometer (ALC‐ET03/06, Shanghai Alcott Biotech Co., Ltd). Blood and liver tissue were harvested 8 hours after LPS injection.

To establish caecal ligation and puncture (CLP)‐induced sepsis mouse model, C57BL/6J mice were completely anesthetized with 1% pentobarbital solution and a midline abdominal incision was performed. The cecum was ligated at the 1/2 of the distal end and was perforated by sterile needles (7^#^) to induce polymicrobial peritonitis and sepsis. The abdominal wall was sutured in two layers followed by injection of 0.1 mL saline subcutaneously for fluid resuscitation. The animals in the sham group underwent laparotomy and bowel manipulation without ligation and perforation. Wogonin treatment was performed 2 hours before CLP operation. Sham and CLP groups were treated with vehicle at the same time. Body temperature and respiratory rate were monitored 2 hours before and 12 hours after operations. Blood, liver and kidney tissue were harvested 12 hours after CLP operation.

### 2.2 Cell culture

Mouse hepatic cell line AML12 was purchased from ATCC, which was cultured in DMEM/F‐12 medium (Gibco) supplemented with 10% foetal bovine serum (FBS, Invitrogen), ITS‐X (Insulin‐Transferrin‐Selenium‐Ethanolamine, Gibco) and 40 ng/ml dexamethasone (Beyotime Biotechnology, Jiangsu, China). The cell was incubated in humidified atmosphere of 5% CO_2_ at 37°C. For the establishment of septic liver injury model in vitro, AML12 cells were treated with 200 ng/mL LPS and 100 ng/mL recombinant mouse TNF‐α (BioLegend Inc) for 6 hours. The cells were harvested for further studies. The cell line was tested for mycoplasma contamination with negative results.

### 2.3 Detection of cytokines and hepatic transaminases by ELISA

Detection of TNF‐α, IL‐1β, interferon‐γ (IFN‐γ) and IL‐6 in serum was performed using the commercially available ELISA kits (Dakewe Bio‐engineering Co, Ltd, Cat. No: 1217202 for TNF‐α, 1210122 for IL‐1β, 1210002 for IFN‐γ, 1210602 for IL‐6). Detection of aspartate transaminase (AST) and alanine transaminase (ALT) in serum was performed using the pre‐coated ELISA kits (mlbio, Cat. No: ml002169‐1 for ALT, ml058577‐1 for AST). Blood samples were collected 8 hours after LPS i.p. whereas 12 hours after CLP operation, centrifuged at 1200 *g* for 20 minutes at 4°C, and the supernatant was collected to detect TNF‐α, IL‐1β, IL‐6, IFN‐γ, AST and ALT levels in serum. All the procedures were done according to the manufacturers’ instructions. The plates were measured with an ELx 808 plate reader (Bio‐Tek Instruments Inc) at 450 nm.

### 2.4 Oxidative stress‐associated assays

Malondialdehyde (MDA) level was detected with Lipid Peroxidation MDA Assay Kit (Beyotime, S0131M) according to the manufacturers’ instruction. In brief, liver tissue and hepatocytes were lysed by RIPA buffer (Beyotime) added with PMSF (Beyotime) for 30 minutes, centrifuged with 12 000 *g* at 4°C for 30 minutes and the supernatant was collected and the protein concentration was measured by the bicinchoninic acid (BCA) method kit (Solarbio). Thiobarbituric acid (TBA) working solution and standard curves were prepared, followed by mix of 0.1 mL supernatant and the working solution. The mixture was heated by boiling water for 15 minutes, cooled down to room temperature and centrifuged at 1000 *g* for 10 minutes. About 0.2 mL of the supernatant was collected, transferred to 96‐well plate and measured with the ELx 808 plate reader (Bio‐Tek Instruments Inc) at 532 nm.

SOD activity was detected with Total Superoxide Dismutase Assay Kit (Beyotime, S0101M) according to the manufacturers’ instruction. In brief, the liver was perfused by saline with 0.16 mg/mL heparin sodium at first and was homogenized with pre‐cooled PBS on ice. The cells were collected, washed twice and homogenized with pre‐cooled PBS. The homogenate was centrifuged with 12 000 *g* at 4°C for 30 minutes, and the supernatant was collected. The protein concentration was measured by BCA method. The supernatant containing 100 μg protein was collected and diluted (1:20) for detection. Working solution and detection buffer was prepared according to the instruction, followed by mixing with samples in 96‐well plate. The mixture was incubated at 37°C for 30 minutes and measured with the ELx 808 plate reader at 450 nm. Inhibitory rate% (I%) was calculated as (A_NC1_ − A_sample_)/(A_NC1_ − A_NC2_) × 100% (A, absorbance at 450 nm, NC, negative control wells). SOD activity was calculated as I%/(1 − I%) for statistical analysis.

Intracellular ROS detection was performed by flow cytometry with the indicator 5‐(and‐6)‐chloromethyl‐2′,7′‐dichlorodihydrofluorescein diacetate acetyl ester (CM‐H_2_DCFDA) (Invitrogen, C6827) according to the manufacturers’ instruction. In brief, ROS indicator (50 μg/tube) was dissolved with 30 μL DMSO for storage. The cells were collected, centrifuged 240 *g* for 5 minutes, washed twice with PBS and resuspended with 100 μL DMEM/F‐12 medium including 10 μmol/L indicator without FBS. The mixture was incubated at 37°C for 30 minutes protected from light, followed by being washed with DMEM/F‐12 medium without FBS twice and resuspended for flow cytometry detection (Beckman Coulter) using excitation sources and filters appropriate for fluorescein isothiocyanate (FITC).

### 2.5 Haematoxylin and Eosin (H&E) staining

Liver and kidney tissue of the sepsis mice were harvested, immersed in 4% paraformaldehyde fixative and then embedded in paraffin. Fixed sections (4 μm‐thick) were stained with haematoxylin‐eosin reagents (Sigma‐Aldrich) and finally observed with a light microscopy (Olympus). For statistical analysis, 2 fields (magnification, 200×) were observed from one slide for scoring. The liver histopathological scores were calculated as previously reported.^18^ The renal tissue damage scores were calculated as previously reported.^19^ Briefly, the percentage of damaged renal tubules was used to assess the score of tissue damage: 0, no damage; 1, <25% damage; 2, 25%‐50% damage; 3, 51%‐75% damage; 4, >75% damage, and the criteria of tubular damage included loss of brush border, tubular dilation, cast formation, inflammatory cell infiltration and cell lysis.

### 2.6 Immunohistochemical staining

Liver tissue of the sepsis mice for immunohistochemical staining (IHC) staining was harvested, immersed in 4% paraformaldehyde fixative and then embedded in paraffin and sectioned into 4 μm‐thick slices. The subsequent steps accomplished with biotin‐streptavidin peroxidase method (SPlink Detection Kit, ZSGB‐Bio) were performed according to the manufacturer's instruction. In brief, the paraffin‐embedded slides were deparaffinized, rehydrated with graded ethanol dilutions, subjected to antigen retrieval, incubated with 30% H_2_O_2_‐CH_3_OH (30% H_2_O_2_ 50 mL, ddH_2_O 50 mL and CH_3_OH 400 mL) to inactivate endogenous peroxidases, blocked with goat serum at room temperature for 20 minutes, respectively. The slides were incubated with the corresponding primary antibodies at 4°C overnight, which were washed with PBS, followed by the incubation with biotinylated goat anti‐rabbit IgG and subsequent HRP‐conjugated streptomycin. Diaminobenzidine (ZSGB‐Bio) was added to the slides for chromogenic reaction. The slides were mounted and observed with optical microscope (Olympus).

The primary antibodies used for IHC staining were against Nrf2 (rabbit polyclonal, ProteinTech, 16396‐1‐AP, 1:100), HO‐1 (rabbit polyclonal, Affinity Biosciences, AF5393, 1:100), SOD1 (rabbit polyclonal, ProteinTech, 10269‐1‐AP, 1:100), SOD2 (mouse monoclonal, ProteinTech, 66474‐1‐Ig, 1:100) and p‐p65 (Ser536) (rabbit polyclonal, Affinity Biosciences, AF2006, 1:100). The standard of staining scores was described previously.^20^ In brief, the percentages of staining‐positive cells were evaluated into four categories: 0 (0%), 1 (1%‐33%), 2 (34%‐66%) and 3 (67%‐100%). The staining intensities were evaluated into four grades: 0 (none), 1 (week), 2 (moderate) and 3 (strong). The final staining score was defined as the product of the percentage and intensity scores.

### 2.7 TUNEL staining

Liver tissue of the sepsis mice for TUNEL staining was harvested, immersed in 4% paraformaldehyde fixative and then embedded in paraffin and sectioned into 4 μm‐thick slices. TUNEL staining was performed with the use of One Step TUNEL Apoptosis Assay Kit (Beyotime, C1088) according to the manufacturer's instructions. In brief, the paraffin‐embedded slides of liver tissue were deparaffinized, rehydrated with graded ethanol dilutions, incubated with 20 μg/mL Proteinase K (Beyotime, ST533) in Immunol Staining Wash Buffer (Beyotime, P0106) at 37°C for 30 minutes and washed by PBS three times. TUNEL working solution was prepared according to the instruction, and the slices were incubated with 50 μL TUNEL working solution at 37°C for 60 minutes, subsequently DAPI (Beyotime) for 15 minutes protected from light, washed by PBS for three times and observed with fluorescence microscope (Olympus) at 488 nm (excitation wavelength). For statistical analysis, 2 fields (magnification, 100×) were observed from one slide and TUNEL positive cells were counted for comparison, as the previous study reported.^21^

### 2.8 RNA extraction and qRT‐PCR

Total RNA was extracted using EZNA Total RNA Kit II (OMEGA Bio‐tek) according to the manufacturer's instructions. Reverse transcription was performed using PrimeScript RTase (Takara Bio Inc) according to the manufacturer's protocol. The expression levels of TNF‐α, IL‐1β, IL‐6 mRNA in liver tissue and AML12 cell lines were determined with real‐time quantitative reverse transcription PCR (qRT‐PCR) using Premix Ex Taq (Takara) according to the manufacturer's instructions and normalized to the expression levels of the endogenous control, β‐actin. The cycling conditions were as following: 95°C for 2 minutes followed by 40 cycles of denaturation at 95°C for 5 seconds, annealing at 55°C for 10 seconds and extension at 72°C for 45 seconds. All reactions were run in triplicate. The resulting amplification and melt curves were analysed to ensure the identity of the specific PCR product. Threshold cycle values were used to calculate the fold change in the transcript levels by using the 2^−ΔΔCt^ method. The primers used for qRT‐PCR were listed in Table S1.

### 2.9 Immunoblotting and antibodies

AML12 cells were lysed using RIPA buffer (Beyotime) added with PMSF (Beyotime). Protein concentration was measured using the BCA method kit (Solarbio). Protein samples were separated by 10% SDS‐PAGE (Beyotime) and then transferred to polyvinylidene fluoride membranes (PVDF, Millipore). After blocking with 5% non‐fat milk for 1 hour, the membrane was incubated with primary antibodies at 4°C overnight and with the corresponding horse radish peroxidase (HRP)‐conjugated secondary antibody (1:2000 dilution) for 1 hour at room temperature the next day. Finally, the blots were detected using enhanced chemiluminescence substrate (ECL kit, Millipore). The phosphorylated protein was normalized to the corresponding total protein. The primary antibodies used for immunoblotting were against Nrf2 (rabbit polyclonal, ProteinTech, 16396‐1‐AP, 1:1000), p65 (rabbit polyclonal, ProteinTech, 10745‐1‐AP, 1:2000), p‐p65 (Ser536) (rabbit monoclonal, Cell Signaling Technology, #3033, 1:1000), β‐actin (mouse monoclonal, Cell Signaling Technology, #3700, 1:3000), NQO‐1 (rabbit polyclonal, Affinity Biosciences, DF6437, 1:500), GST‐M1 (rabbit polyclonal, ProteinTech, 12412‐1‐AP, 1:500), HO‐1 (rabbit polyclonal, Affinity Biosciences, AF5393, 1:500), SOD1 (rabbit polyclonal, ProteinTech, 10269‐1‐AP, 1:1000), SOD2 (mouse monoclonal, ProteinTech, 66474‐1‐Ig, 1:1000), Caspase3 (mouse monoclonal, ProteinTech, 66470‐2‐Ig, 1:1000), Cleaved‐Caspase3 (rabbit monoclonal, Cell Signaling Technology, #9664, 1:1000), Bax (rabbit polyclonal, ProteinTech, #50599‐2‐Ig, 1:2000), Bcl‐2 (rabbit polyclonal, ProteinTech, #12789‐1‐AP, 1:1000), PARP (rabbit monoclonal, Cell Signaling Technology, #9532, 1:1000), Cleaved‐PARP (Asp214) (rabbit monoclonal, Cell Signaling Technology, #5625, 1:500) and the second antibodies were HRP‐linked goat anti‐rabbit antibody (Cell Signaling Technology, #7074, 1:2000), HRP‐linked goat anti‐mouse antibody (Cell Signaling Technology, #7076, 1:2000).

### 2.10 Immunofluorescence staining

AML12 cells were grown and treated on cell culture dish with glass bottom. After the treatment, the cells were washed by PBS, fixed by paraformaldehyde, blocked by the goat serum and were incubated with primary antibody (Nrf2, rat monoclonal, CST, #14596, 1:50) at 4°C overnight. The cells were subsequently incubated with the secondary antibody (Alexa Fluor 488 Donkey anti‐Rat IgG (H + L), Invitrogen, A21208) for 2 hours and the nuclear dye DAPI (Beyotime) for 15 minutes at room temperature. Fluorescent images were obtained by confocal microscope (Nikon), and statistical analysis was performed according to the previous study.^22^

### 2.11 RNA interference

Small interfering RNA (siRNA) specifically targeting Nrf2 (siNrf2) and negative control siRNA (siNC) were designed and synthesized by GenePharm. Sequences of the siRNA were listed in Table S2. The siRNA was transfected into cells using Lipofectamine™ 3000 (Invitrogen) and Opti‐MEM (Gibco). The transfection procedure was performed according to the manufacturer's instructions.

### 2.12 Flow cytometry analysis of cell apoptosis

Cell apoptosis was detected with Annexin V‐FITC/PI Apoptosis Detection Kit (Beyotime, C1062L) according to the manufacturers’ instruction. In brief, AML12 cells were harvested, washed twice with 4°C PBS and re‐suspended in binding buffer with Annexin V‐FITC staining agent of the kit. After incubation at 37°C for 30 minutes, propidium iodide (PI) was added to the stain cells 15 minutes before the analysis by flow cytometry.

### 2.13 Detection of blood urea nitrogen (BUN) and creatinine

BUN and creatinine in serum were detected to evaluate renal dysfunction. Blood samples were collected 12 hours after CLP operation, centrifuged at 4000 rpm for 20 minutes at 4°C, and the supernatant was collected to detect BUN and creatinine levels using commercial kits (Jiancheng Bioengineering Institute, Nanjing, China, Cat. No: C013‐2‐1 for BUN and C011‐2‐1 for creatinine). All the procedures were done according to the manufacturers’ instructions. The plates were measured with an ELx 808 plate reader (Bio‐Tek Instruments Inc) at 640 nm for BUN and 546 nm for creatinine.

### 2.14 Statistical analysis

The results were analysed by two‐tailed Student's *t* test or one‐way analysis of variance (ANOVA). The survival curves were analysed by log‐rank (Mantel‐Cox) test. The results were presented as mean ± SD through at least 3 independent experiments, with *P* < .05 considered to be statistically significant. Statistical analyses were accomplished using GraphPad Prism 6.0 (GraphPad Software).

## 3 RESULTS

### 3.1 Wogonin treatment improves the prognosis of mice with LPS‐ or CLP‐induced sepsis

**FIGURE 1:**
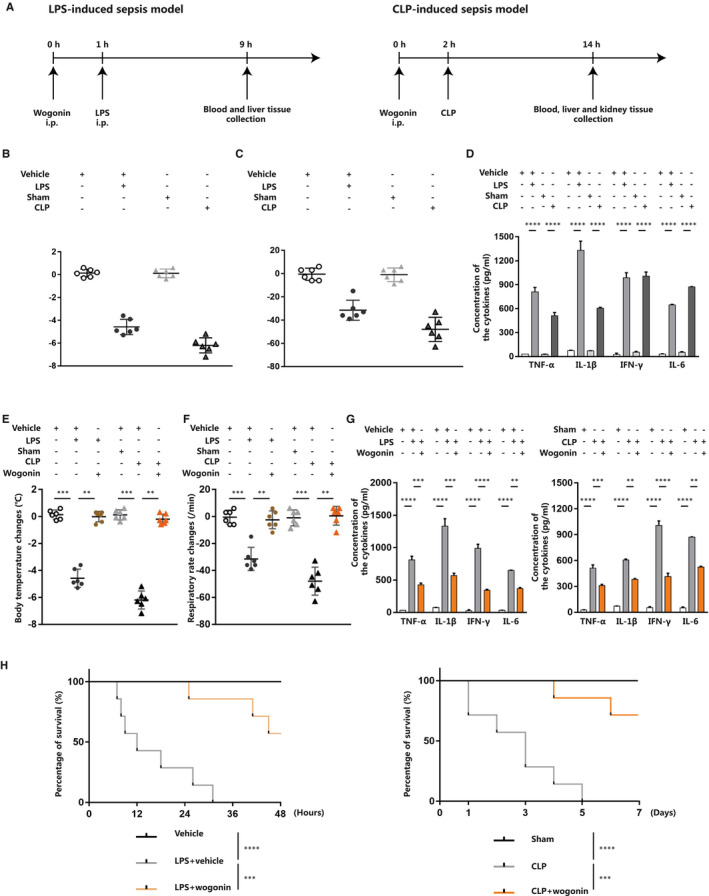
Wogonin treatment improves the prognosis of mice with LPS‐ or CLP‐induced sepsis. A, Schematic presentation of the establishment of LPS‐ and CLP‐induced sepsis mouse model. B and C, Body temperature and respiratory rate changes 1 h before and 8 h after LPS injection or 2 h before and 12 h after CLP operation in sepsis mouse model (n = 6, respectively). D, Detection of serum TNF‐α, IL‐1β, IFN‐γ and IL‐6 in LPS‐ and CLP‐induced sepsis mice (n = 6). E and F, Body temperature and respiratory rate changes after wogonin treatment in LPS‐ and CLP‐induced sepsis mice (n = 6). G, Detection of serum TNF‐α, IL‐1β, IFN‐γ and IL‐6 after wogonin treatment in LPS‐ and CLP‐induced sepsis mice (n = 6). H, Survival analysis of LPS‐ and CLP‐induced sepsis mice (n = 7) with or without wogonin treatment. The concentration of wogonin was 50.0 mg/kg. The results are presented as mean ± SD through at least 3 independent experiments and were analysed by one‐way analysis of variance (ANOVA). The results of survival comparison were analysed by Kaplan‐Meier survival curves and log‐rank test (Mantel‐Cox method). *P < .05, **P < .01, ***P < .001, ****P < .0001

In order to study whether wogonin exerts its protective role in the treatment of sepsis, we first established the mouse sepsis model via the intraperitoneal injection of LPS or the surgery of caecal ligation and puncture (CLP) as previously described^23^, ^24^ (Figure 1A). In consistent with previous reports, both LPS injection and CLP could effectively induce the phenotype of sepsis in mice, as manifested by prominent decrease of body temperature and respiratory rate^25^ (Figure 1B,C). According to previous studies, increased levels of circulating cytokines like TNF‐α, IL‐1β and IFN‐γ are typical characteristics of sepsis, which may induce endothelium dysfunction, pathological procoagulant state and exacerbate liver injury as well.^26^, ^27^ Besides, the increase of IL‐6 in circulation can also greatly contribute to the onset and progression of sepsis.^27^ Therefore, these pro‐inflammatory cytokines in serum are critical pathological factors and valuable indicators of sepsis. Our results showed that either LPS injection or CLP surgery significantly up‐regulated the levels of circulating TNF‐α, IL‐1β, IFN‐γ and IL‐6 in mice (Figure 1D), indicating successful establishment of sepsis mouse model.

To investigate the effect of wogonin on the outcome of sepsis mice, we intraperitoneally injected LPS‐ or CLP‐induced sepsis mice with different dose of wogonin. As was shown, wogonin treatment was capable of reversing the decreased body temperature and respiratory rate caused by LPS injection or CLP surgery (Figure 1E,F and Figure S1A,B). In addition, the concentrations of pro‐inflammatory cytokines TNF‐α, IL‐1β, IFN‐γ and IL‐6 in circulation were prominently repressed after wogonin treatment in these two types of sepsis mice (Figure 1G), with the levels of these cytokines gradually decreased as the dose of wogonin increased (Figure S1C,D). Of note, the lethality is reported to occur in an acute manner in LPS‐induced sepsis mouse model, whereas begins relatively later after the surgery of CLP,^24^, ^28^ as was revealed in previous reports and our Kaplan‐Meier survival analysis (Figure 1H). We further illustrated the survival rate of LPS‐ or CLP‐induced sepsis mice 48 hours or 7 days after wogonin treatment, respectively, which showed that wogonin treatment prominently improved the survival of these two types of sepsis mice (Figure 1H), in particular in a dose‐dependent manner (Figure S1E,F). Taken together, our results demonstrate that wogonin was able to effectively improve the manifestations, outcome of mice with LPS‐ or CLP‐induced sepsis and suppress the levels of circulating pro‐inflammatory cytokines.

### 3.2 Wogonin mitigates liver injury in mice with sepsis

**FIGURE 2:**
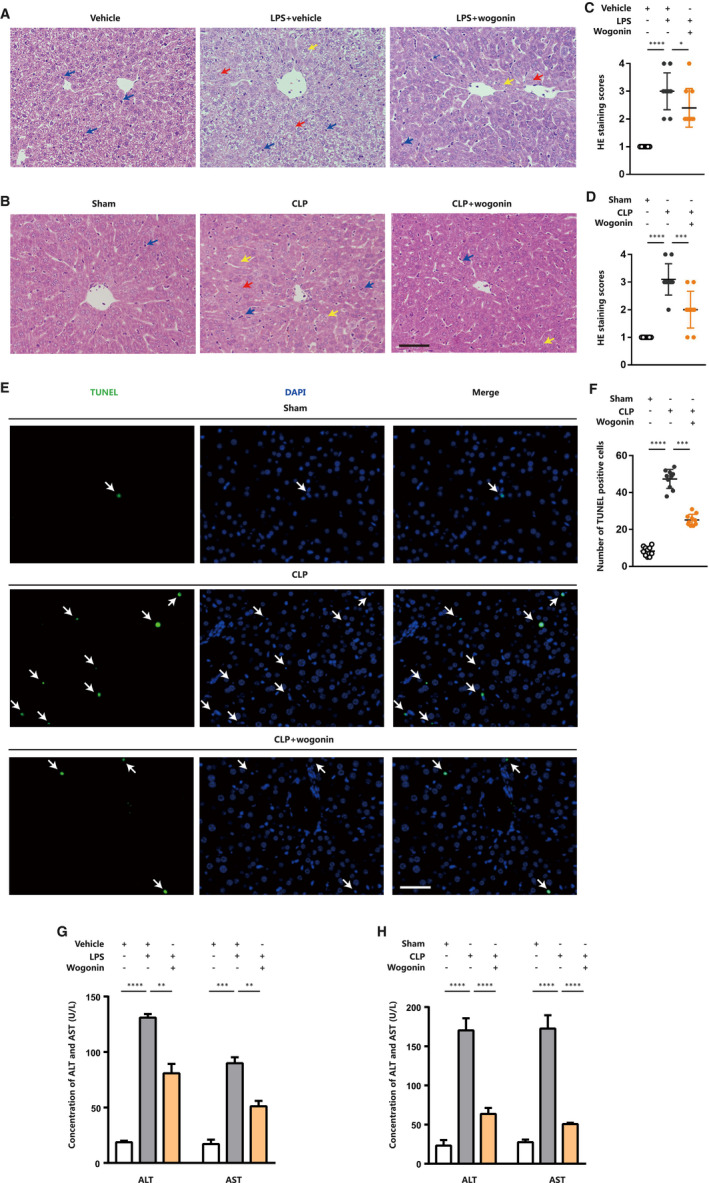
Wogonin mitigates liver injury in mice with LPS‐ or CLP‐induced sepsis. A and B, Representative H&E staining images of liver tissue from LPS‐ and CLP‐induced sepsis mice (n = 5). The red arrows indicate obscure nucleus of hepatocytes. The yellow arrows indicate hepatocytes with vacuolar degeneration. The blue arrows indicate infiltrated neutrophils or macrophages. Magnification, 200×. Scale bar = 50 μm. C and D, H&E staining scores of liver injury in LPS‐ and CLP‐induced sepsis mice (n = 5). The number of scored fields = 10. E, Representative TUNEL staining images of liver tissue in CLP‐induced sepsis mice (n = 5). The arrows indicate the TUNEL‐positive cells. Magnification, 200×. Scale bar = 50 μm. F, The number of TUNEL‐positive cells in the liver of CLP‐induced sepsis mice with or without wogonin treatment (n = 5). The number of analysed fields = 10 (magnification, 100×). G and H, Liver injury biomarkers ALT and AST in serum of LPS‐ and CLP‐induced sepsis mice (n = 6). The concentration of wogonin was 50.0 mg/kg. Data are presented as mean ± SD through at least 3 independent experiments and were analysed by one‐way analysis of variance (ANOVA). *P < .05, **P < .01, ***P < .001, ****P < .0001

Sepsis is commonly characterized by the dysfunction of multiple organs, among which the damage of liver occurs frequently and can develop to fulminant hepatitis or a lethal syndrome of hepatocellular, metabolic and hemodynamic disorder as liver failure.^27^, ^29^ Therefore, septic liver injury has been documented as a crucial and independent indicator of the mortality of patients with sepsis.^29^ We therefore went on to investigating the effects of wogonin on liver in sepsis mice. Previous studies have demonstrated the histological malformation of liver tissue in sepsis mice, with different characteristics indicating distinct degrees of liver injury, which were used as histological scoring standard to evaluate the severity of liver injury. To be specific, swollen hepatocytes, disappearance of sinus hepaticus and slightly inflammatory cell infiltration suggest lower degree of liver injury, whereas obscure nucleus, vacuolar degeneration, ballooning degeneration of hepatocytes and severe inflammatory cell infiltration indicate moderate degree of liver injury. In addition, the above‐mentioned alterations of moderate degree together with focal necrosis indicate higher degree of liver injury.^30^, ^31^ Through the H&E staining analysis, we found that liver tissue from the two sepsis models showed obscure nucleus (indicated by red arrows) and vacuolar degeneration (indicated by yellow arrows) in hepatocytes (Figure 2A,B). Excessive periportal neutrophil and macrophage infiltration (shown by blue arrows) were also found in liver tissue of LPS‐ and CLP‐induced septic mice (Figure 2A,B). In contrast, the liver tissue isolated from wogonin‐treated sepsis mice displayed mild histological malformation including swollen cells, obscure nucleus and vacuolar degeneration, as well as less infiltration of inflammatory cells (Figure 2A,B). In line with the histological appearance, the staining scores were significantly higher in liver tissue of either LPS‐ or CLP‐induced sepsis mice compared with the control, whereas wogonin treatment could re‐suppress the increased histological scores, indicating ameliorated septic liver injury (Figure 2C,D). In line with this, our TUNEL staining showed that wogonin treatment prominently reduced the apoptotic rate of hepatocytes in liver tissue from these two types of sepsis mice (Figure 2E,F and Figure S2A,B). The biomarkers ALT and AST in serum for reflecting liver injury were both prominently increased in sepsis mice, whereas significantly re‐suppressed after wogonin treatment (Figure 2G,H), forwardly supporting that wogonin could reduce the severity of liver injury in sepsis mice.

Considering the protective role of wogonin reported in drug‐ or heavy metal‐induced kidney injury,^13^, ^14^ we also investigated the effect of wogonin on CLP‐induced kidney injury. To evaluate the effect of wogonin on renal dysfunction, we examined levels of BUN and serum creatinine 12 hours after CLP operation (Figure S2C,D). The results showed that CLP operation caused significant increase of BUN and creatinine, and wogonin treatment efficiently reduced both of the BUN and creatinine levels, indicating the therapeutic effect of wogonin on CLP‐induced septic kidney dysfunction. Besides, we conducted H&E staining analysis of renal tissues to examine the histological malformation of renal tissue in CLP‐induced sepsis mice. According to the previous study,^19^ the percentage of damaged renal tubules was used to assess the score of tissue damage. The criteria of tubular damage included loss of brush border, tubular dilation, cast formation, inflammatory cell infiltration and cell lysis. Through H&E staining analysis, we found that renal tissue from CLP‐induced sepsis mice showed more severe characteristics of renal histological damage, including cell necrosis or lysis (indicated by red arrows), cast formation (indicated by yellow arrows) and inflammatory cell infiltration (indicated by blue arrows) than the sham group (Figure S2E). In contrast, the renal tissue from wogonin‐treated sepsis mice displayed mild histological malformation (Figure S2E). In line with the histological observation, the staining scores were significantly higher in renal tissues in CLP‐induced sepsis mice compared with the sham group, whereas wogonin treatment could reduce the score of renal tissue damage, indicating ameliorated CLP‐caused renal injury induced by wogonin treatment (Figure S2F).

### 3.3 Wogonin activates Nrf2 pathway and alleviates oxidative stress in hepatocytes

**FIGURE 3:**
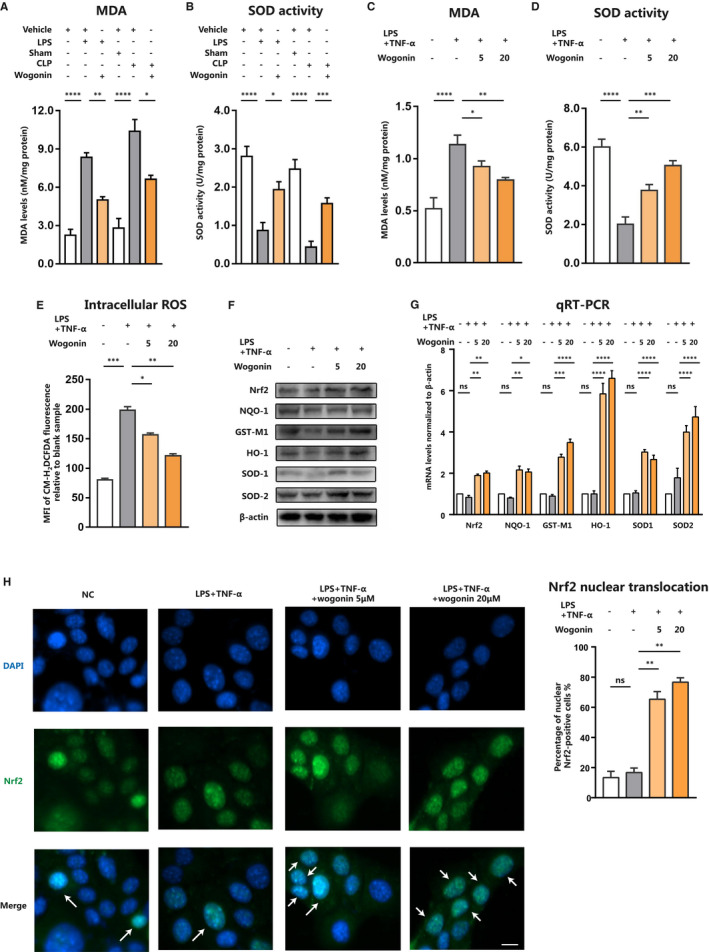
Wogonin activates Nrf2 pathway and alleviates oxidative stress in hepatocytes. A and B, MDA and SOD activity in liver tissue from LPS‐ or CLP‐induced sepsis mice with or without wogonin treatment (n = 6). C and D, MDA and SOD activity in LPS/TNF‐α‐stimulated AML12 cells with or without wogonin treatment. E, Intracellular ROS analysed by flow cytometry in AML12 cells. MFI, the mean fluorescence intensity. F and G, Protein and mRNA levels of Nrf2 and downstream antioxidant enzymes NQO‐1, GST‐M1, HO‐1, SOD1 and SOD2 in LPS/TNF‐α stimulated AML12 cells with or without wogonin treatment. H, Representative images of Nrf2 nuclear translocation detected by immunofluorescence staining and the percentage of nuclear Nrf2‐positive cells in the cells with indicated treatment. The arrows show the Nrf2 nuclear positive cells. Magnification, 200×. Scale bar = 10 μm. NC, the negative control group. The results are presented as mean ± SD through at least 3 independent experiments and were analysed by one‐way analysis of variance (ANOVA). ns, no statistical significance, *P < .05, **P < .01, ***P < .001

Accumulative evidence has revealed that oxidative stress plays a crucial role in the pathogenesis of septic liver injury. The excessive generation of ROS could not only directly cause the cell death of hepatocytes by augmenting the formation of lipid peroxides and oxidative protein adducts, but also amplify the pro‐inflammatory signalling cascades to induce more severe systematic immune and inflammatory responses.^32^, ^33^, ^34^, ^35^ Therefore, we turned to study whether wogonin exerted its therapeutic effect on septic liver injury via the prevention of oxidative stress. As predicted, LPS injection or CLP surgery promoted the formation of lipid peroxidation product MDA in liver tissue of sepsis mice. Meanwhile, the activity of the crucial anti‐oxidative enzyme SOD in liver was prominently repressed (Figure 3A,B). After the treatment with wogonin, the accumulation of MDA in liver was significantly ameliorated, together with the increased activity of SOD (Figure 3A,B), indicating the potent anti‐oxidative capacity of wogonin in vivo. Previous reports have revealed that the exposure to LPS combined with TNF‐α mimics the stressful condition accounting for liver inflammatory response and liver injury in sepsis.^36^, ^37^, ^38^, ^39^ We cultured AML12 hepatocytes in vitro and stimulated them with LPS combined with TNF‐α, which induced significant increase of MDA and decrease of SOD activity (Figure 3C,D). Consistent with the effect in vivo, wogonin treatment could also prominently suppress the accumulation of MDA and recover the activity of SOD in vitro (Figure 3C,D), so was the generation of intracellular ROS detected by flow cytometry stained with CM‐H_2_DCFDA (Figure 3E). These results provide the evidence that wogonin exerted potent anti‐oxidative capacity in septic liver injury.

Nrf2 is a master regulator of anti‐oxidative machinery in various tissues and cells. Accumulating evidence has revealed that wogonin was capable of activating Nrf2 pathway to exert anti‐oxidative effect, coupling with anti‐inflammatory capacity. In addition, to activate Nrf2 signalling could ameliorate acute liver injury by facilitating the expressions of downstream anti‐oxidant enzymes so as to restrain oxidative stress.^40^, ^41^ Therefore, we proposed that wogonin might play the protective role in septic liver injury via the activation of Nrf2. Given that Nrf2/ARE signalling transcriptionally activates antioxidant enzymes like HO‐1, NAD(P)H:quinone oxidoreductase (NQO‐1), glutathione S‐transferase (GST), superoxide dismutase SOD1 and SOD2 to mitigate oxidative stress‐induced damage,^7^, ^42^, ^43^ we thus detected the expressions of these target genes. Through the immunoblotting analysis, we discovered that wogonin treatment abolished the suppressed expressions of Nrf2 and its downstream NQO‐1, GST‐M1, HO‐1, SOD1 and SOD2 caused by LPS combined with TNF‐α (Figure 3F). Subsequent qRT‐PCR assay revealed the similar alteration trend as immunoblotting analysis showed, suggesting the potentiated transcriptional activity of Nrf2 after the wogonin treatment (Figure 3G). Furthermore, through the immunofluorescence staining analysis, we found that wogonin treatment promoted the nuclear translocation of Nrf2 (Figure 3H), which is essential for its transcriptional activity that promotes the expressions of downstream anti‐oxidative enzymes. Taken together, these results demonstrate that wogonin could alleviate oxidative stress in hepatocytes and effectively activate Nrf2 pathway.

### 3.4 Wogonin prevents hepatocytes from LPS/TNF‐α‐induced apoptosis

**FIGURE 4:**
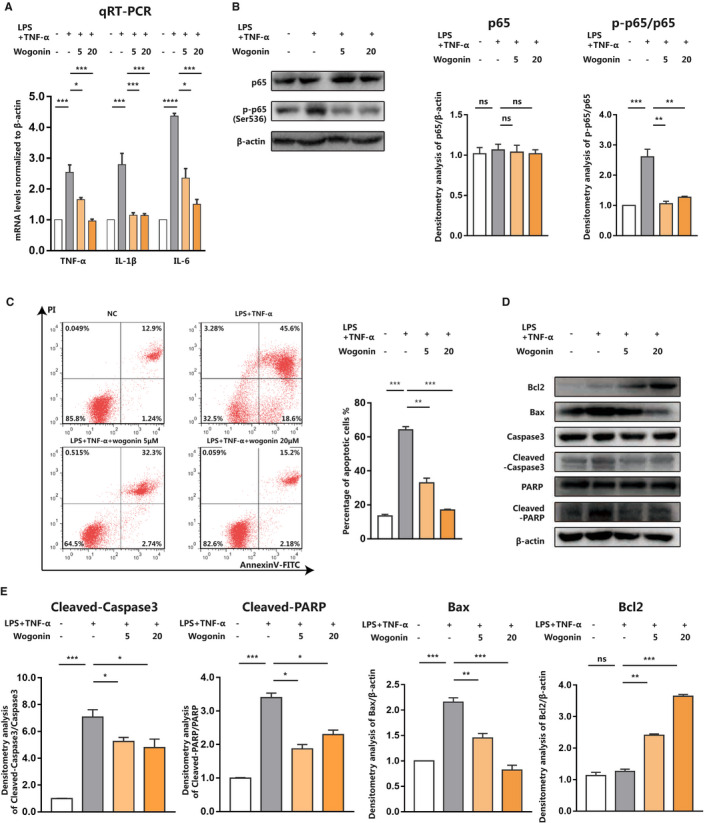
Wogonin prevents hepatocytes from LPS/TNF‐α‐induced apoptosis. A, qRT‐PCR analysis of TNF‐α, IL‐1β and IL‐6 mRNA levels and (B) immunoblotting analysis of p65 and p‐p65 (Ser536) after LPS/TNF‐α and wogonin treatment with indicated concentrations in AML12 cells. C, Representative flow cytometry images and analysis of cell apoptosis after indicated treatment in AML12 cells. D, Immunoblotting analysis of pro‐apoptotic Bax, Cleaved‐Caspase3, Cleaved‐PARP and anti‐apoptotic Bcl2 expression related to (C). E, Densitometry analysis of Cleaved‐caspase3, Cleaved‐PARP, Bax and Bcl2 related to (D). NC, the negative control group. The results are presented as mean ± SD through at least 3 independent experiments and were analysed by one‐way analysis of variance (ANOVA). *P < .05, **P < .01, ***P < .001

It has been unveiled that Nrf2 possesses the anti‐inflammatory capacity that is highly associated with the interplay between Nrf2 signalling and NF‐κB signalling. The genetic deletion of *Nrf2* can potentiate inflammation whereas its up‐regulation suppresses pro‐inflammatory responses controlled by NF‐κB, thus contributing to the progression of chronic obstructive pulmonary disease (COPD), traumatic brain injury and some other diseases.^44^, ^45^, ^46^, ^47^ As we discovered that wogonin was able to activate Nrf2 and alleviate oxidative stress, we wondered whether it could restrain pro‐inflammatory response in sepsis liver. Our qRT‐PCR analysis in AML12 cells showed that while LPS combined with TNF‐α elevated the levels of TNF‐α, IL‐6 and IL‐1β, wogonin treatment prominently suppressed the expressions of these pro‐inflammatory cytokines (Figure 4A). In addition, our immunoblotting analysis revealed that in response to the stimulation with LPS combined with TNF‐α, the phosphorylation of NF‐κB was significantly increased, whereas wogonin treatment could restrain the activation of NF‐κB (Figure 4B). These results indicated the great potential of wogonin in the prevention of inflammatory response in sepsis liver.

Our in vitro experiments in AML12 cells proved that wogonin could simultaneously potentiate the activity of Nrf2‐dependent anti‐oxidative machinery and inhibit NF‐κB‐dependent pro‐inflammatory pathway. Thereafter, we examined whether wogonin could protect hepatocytes against septic liver injury in vitro as well. The stimulation of LPS combined with TNF‐α resulted in robust increase of apoptosis of AML12 cells, which was significantly suppressed after the co‐treatment with wogonin, especially in a dose‐dependent manner (Figure 4C). Moreover, we employed immunoblotting analysis to testify the expressions of apoptosis‐related molecules. The expression of anti‐apoptotic Bcl2 was markedly increased, whereas the expressions of pro‐apoptotic Bax, Cleaved‐Caspase3 and Cleaved‐PARP were significantly decreased after the co‐treatment with wogonin (Figure 4D,E). Combined with the results in sepsis mice, our data proved that wogonin could ameliorate septic liver injury both in vitro and in vivo.

### 3.5 Wogonin ameliorates liver injury in a Nrf2‐dependent manner in sepsis

**FIGURE 5:**
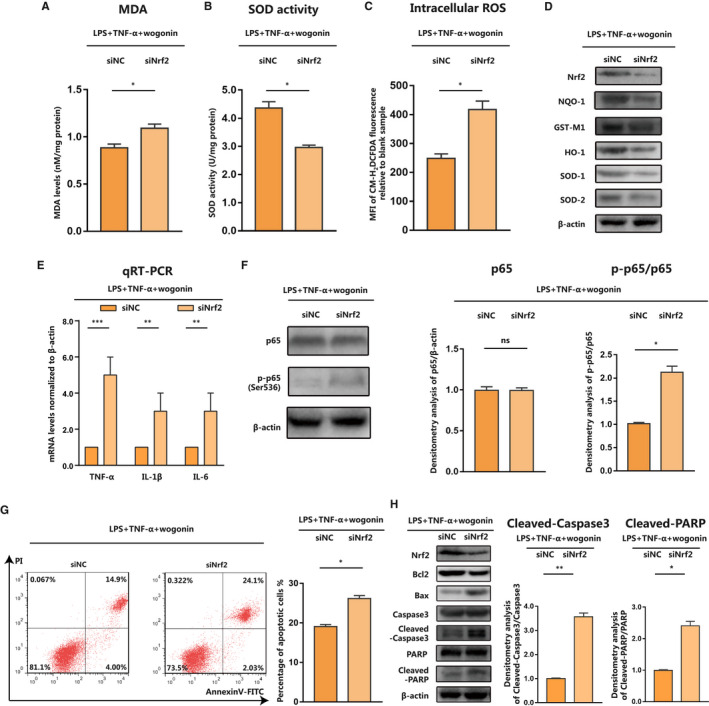
Wogonin ameliorates septic liver injury in a Nrf2‐dependent manner. A and B, MDA and SOD activity in AML12 cells with indicated treatments. C, Intracellular ROS analysed by flow cytometry and represented as MFI of CM‐H_2_DCFDA in AML12 cells. D, Immunoblotting analysis of Nrf2, NQO‐1, GST‐M1, HO‐1, SOD1 and SOD2 in AML12 cells with indicated treatments. E and F, qRT‐PCR analysis of TNF‐α, IL‐1β and IL‐6 mRNA levels and immunoblotting analysis of p65 and p‐p65 (Ser536) in AML12 cells with indicated treatments. G, Representative flow cytometry images and analysis of cell apoptosis in AML12 cells with indicated treatments. H, Immunoblotting analysis of Nrf2, pro‐apoptotic Bax, Cleaved‐Caspase3 and Cleaved‐PARP together with anti‐apoptotic Bcl2 expression and densitometry analysis of Cleaved‐caspase3 and Cleaved‐PARP. The concentration of wogonin is 10 μmol/L. The results are presented as mean ± SD through at least 3 independent experiments and were analysed by two‐tailed Student's *t* test or one‐way analysis of variance (ANOVA). ns, no statistical significance, *P < .05, **P < .01, ***P < .001

Forwardly, we went on to investigating whether wogonin exerted its protective effects on septic liver injury via the activation of Nrf2. We transfected AML12 cells with siRNA against Nrf2 to obtain the knock‐down of Nrf2 expression, followed by the treatment with LPS combined with TNF‐α and wogonin. As was shown, the knock‐down of Nrf2 abolished the suppressive effect on MDA accumulation of wogonin in LPS/TNFα‐stimulated AML12 cells (Figure 5A). In addition, the activity of anti‐oxidative SOD potentiated by wogonin was also re‐suppressed after the knock‐down of Nrf2 (Figure 5B), whereas the accumulation of intracellular ROS was potentiated by the knockdown of Nrf2 (Figure 5C). Subsequent immunoblotting analysis revealed that Nrf2 expression deficiency abolished the facilitative role of wogonin in the up‐regulation of anti‐oxidative enzymes including NQO‐1, GST‐M1, HO‐1, SOD1 and SOD2 (Figure 5D). Moreover, the deficiency of Nrf2 expression re‐amplified the transcriptional levels of pro‐inflammatory cytokines TNF‐α, IL‐6 and IL‐1β (Figure 5E), so was the phosphorylation of NF‐κB p65 subunit (Figure 5F). Furthermore, the knock‐down of Nrf2 expression exacerbated LPS/TNF‐α‐induced apoptosis of AML12 cells (Figure 5G), with the expressions of pro‐apoptotic Bax, Cleaved‐Caspase3 and Cleaved‐PARP increased and anti‐apoptotic Bcl2 decreased (Figure 5H). Taken together, wogonin exerted its protective role in septic liver injury in a Nrf2‐dependent manner via the potentiation of anti‐oxidative capacity and the suppression of pro‐inflammatory cytokines.

### 3.6 Wogonin activates Nrf2 signalling in liver tissue of sepsis mice

**FIGURE 6:**
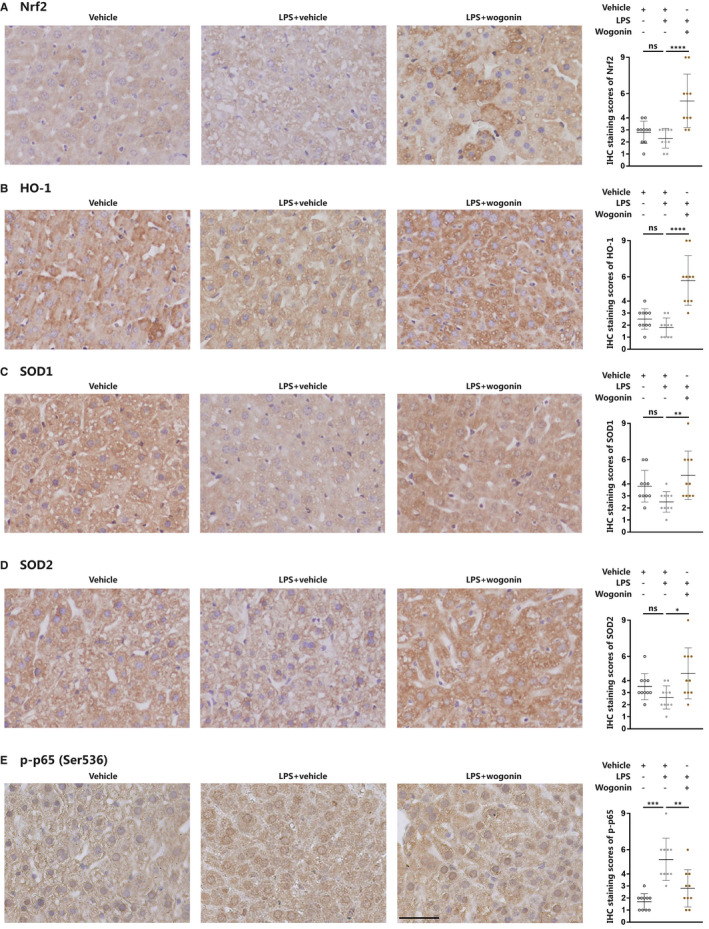
Wogonin activates Nrf2 signalling in liver tissue of LPS‐induced sepsis mice. A‐E, Representative IHC images and IHC score analysis of Nrf2, HO‐1, SOD1, SOD2 and p‐p65 (Ser536) in liver tissue from LPS‐induced septic mice (n = 5). The number of scored fields = 10. Magnification, 400×. Scale bar = 50 μm. The concentration of wogonin was 50.0 mg/kg. The results are presented as mean ± SD through at least 3 independent experiments and were analysed by one‐way analysis of variance (ANOVA). ns, no statistical significance, *P < .05, **P < .01, ***P < .001

Finally, we employed IHC analysis to clarify the role of wogonin in Nrf2 signalling in the liver of sepsis mice. As was revealed, wogonin treatment was able to potentiate the staining scores of Nrf2 and its downstream HO‐1, SOD1 and SOD2 in liver tissue from both LPS‐ and CLP‐induced sepsis mouse models (Figure 6A‐D and Figure S3A‐D). In contrast, the staining score of phosphorylated‐p65 (Ser536) was repressed (Figure 6E and Figure S3E), which was in line with previous in vitro results. Therefore, we provided in vivo evidence that wogonin facilitated the activation of Nrf2 to enhance the anti‐oxidative capacity and restrain the pro‐inflammatory signalling, thus effectively ameliorating septic liver injury.

## 4 DISCUSSION

**FIGURE 7:**
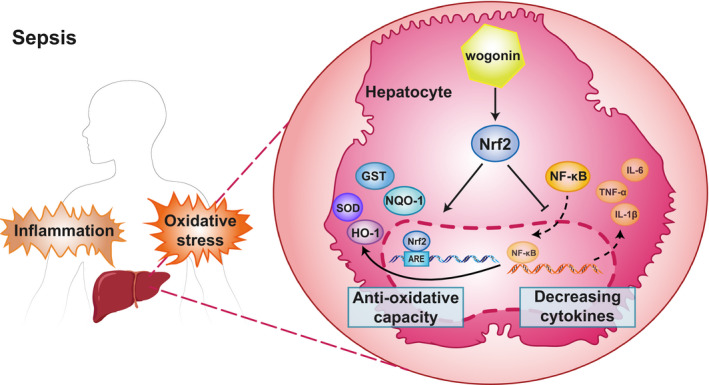
Schematic presentation of the mechanism of wogonin to alleviate septic liver injury. Wogonin treatment activated Nrf2 signalling, promoted transcription and expression of endogenous antioxidants including NQO‐1, GST, HO‐1, SOD which suppressed sepsis‐induced oxidative stress in hepatocytes. Moreover, wogonin‐promoted Nrf2 signalling inhibited NF‐κB activation and decreased production of pro‐inflammatory cytokines, which mitigated inflammatory damage of hepatocytes in sepsis

In the present study, we initially proved that wogonin treatment could improve the survival of mice with LPS‐ or CLP‐induced sepsis, accompanied with the rescue of reduced body temperature and respiratory rate, and the suppression of pro‐inflammatory cytokines TNF‐α, IL‐1β, IFN‐γ and IL‐6 in circulation. Then, our results showed that wogonin was effective in mitigating liver injury in mice with sepsis, which was attributed to the potentiation of anti‐oxidative capacity and the suppression of pro‐inflammatory cytokines. Furthermore, we proved that it was the activation of Nrf2 that was responsible for the protective role of wogonin in liver injury in LPS‐ or CLP‐induced sepsis mice. Finally, we provided in vivo evidence that wogonin activated Nrf2 signalling in liver tissue of sepsis mice. In summary, wogonin is a promising therapeutic agent for preventing liver injury in sepsis mice via the simultaneous regulation of anti‐oxidative machinery and pro‐inflammatory signalling through the activation of Nrf2 (Figure 7).

Previous studies have revealed that oxidative stress plays a critical role in liver injury, because the disturbance of redox balance could induce intracellular dysfunction of biochemical and metabolic processes in hepatocytes.^48^ Moreover, liver is responsible for de‐oxidation reactions, synthesis of lipids and storage of glycogen as the primary metabolic organ, so it is more susceptible to oxidative stress caused by multiple factors including sepsis‐associated inflammatory stimulations. During the progression of sepsis, the generation of ROS is tightly linked to the occurrence and development of liver injury, not only via the direct damage to hepatocytes by promoting the formation of oxidative protein adducts and lipid peroxides, but also through the pro‐inflammatory response accentuated by oxidative stress. Therefore, to ameliorate oxidative stress is a useful strategy to reduce liver injury in sepsis to obtain the improvement of the patients. Our study demonstrated that the generation of ROS in sepsis liver was concomitant with impaired anti‐oxidative capacity. Wogonin treatment was potent in restraining septic liver injury via the potentiation of Nrf2‐dependent anti‐oxidative machinery. Moreover, the alleviation of oxidative stress by wogonin treatment led to suppressed production of pro‐inflammatory cytokines and cell apoptosis in hepatocytes, highlighting the great therapeutic potential of wogonin in oxidative stress‐driven liver injury in sepsis.

As the natural component of *Scutellaria baicalensis* Georgi (also known as Huang Qin), wogonin has been proved as potent activator of Nrf2 so as to exert its regulatory effect on oxidative stress‐related diseases. For example, wogonin played protective role against myocardial infarction by repressing oxidative stress through the activation of Nrf2/HO‐1 pathway.^49^ Besides, wogonin was capable of ameliorating the severity of rheumatoid arthritis and nephrotoxicity resulted from cadmium and cisplatin by lessening oxidative stress.^13^, ^14^, ^50^ Consistent with these reports, our findings further confirmed the potent anti‐oxidative capacity of wogonin in treating septic liver injury via the activation of Nrf2. Therefore, Nrf2 is a crucial mediator of the therapeutic effect of wogonin in many diseases. Extending to previous reports, we also proved that the activation of Nrf2 effectively antagonized the inflammation via the suppression of NF‐κB signalling in sepsis liver, which supports that wogonin was also a potential anti‐inflammatory agent that could be applied for the treatment of inflammatory diseases.

Septic liver injury leads to intrahepatic endothelial dysfunction which facilitates dysregulated molecular transportation between hepatocytes and circulation resulting in systemic metabolism disorder like hyperlipidaemia.^51^, ^52^ Wogonin was found to exert protective role in endothelial cells through inhibition of high mobility group box 1 (HMGB1) in LPS‐ and CLP‐induced sepsis and reduced sepsis‐related mortality in mice.^53^ Therefore, apart from the direct effect on hepatocyte reported in the present study, wogonin can also exert protective effect on septic liver injury by maintaining intrahepatic endothelial barrier, raising the notion that both of the localized hepatocyte and hepatocyte‐associated microenvironment should be taken into consideration in case of the treatment of septic liver injury.

Abnormal activation of NF‐κB signalling plays the pivotal role in inflammatory diseases such as asthma, rheumatoid arthritis and inflammatory bowel disease so that the antagonization of NF‐κB signalling can be an effective therapeutic strategy for inflammatory diseases.^54^ The crosstalk between Nrf2 and NF‐κB signalling pathways has been frequently observed that anti‐oxidative Nrf2 signalling pathway was able to inhibit NF‐κB signalling activation by reducing DNA‐binding activity of NF‐κB.^55^ In respiratory syncytial virus infection model, Nrf2 depletion strengthened DNA‐binding activity of NF‐κB in Nrf2^−/−^ mice and exacerbated inflammation together with more protein/lipid oxidation.^56^ In acute liver injury induced by LPS/D‐GalN injection, Nrf2 inhibited NF‐κB DNA binding by reducing MafK expression, the transcription factor which mediated p65 acetylation and its DNA‐binding affinity.^57^ What's more, as the redox‐sensitive transcriptional factor,^58^ NF‐κB has been proved to be activated by oxidative stress since that intracellular ROS induced the serine phosphorylation of the NF‐κB subunit p65 and thereby facilitated its nuclear translocation and downstream gene expression like IL‐1β.^59^ In Nrf2^−/−^ septic shock mice, NF‐κB signalling was magnified in lung and peritoneal macrophages whereas antioxidants N‐acetyl cysteine and GSH‐monoethyl ester suppressed NF‐κB activation, which implied that anti‐oxidative agents could inhibit NF‐κB signalling by alleviating oxidative stress and re‐suppressed Nrf2 depletion‐induced NF‐κB activation.^60^ The present study consistently showed that suppression of oxidative stress induced by Nrf2 signalling activation restrained p65 phosphorylation and pro‐inflammatory cytokine production, reflecting that anti‐oxidative capacity of wogonin could also attenuate liver injury by suppressing NF‐κB signalling pathway, which suggests that the manipulation of anti‐oxidative stress machinery could be referred as alternative strategy for sepsis and other inflammation‐associated disorders. In conclusion, the present study showed that wogonin could ameliorate septic liver injury and improve the outcome of mice with sepsis via the simultaneous regulation of anti‐oxidative machinery and pro‐inflammatory signalling through the activation of Nrf2 in hepatocytes. Additional clinical trials are needed to confirm the therapeutic potential of wogonin for sepsis in the future.

## CONFLICT OF INTEREST

The authors confirm that there are no conflicts of interest.

## AUTHOR CONTRIBUTION

**Ji‐Min Dai:** Investigation (lead); Writing‐original draft (lead); Writing‐review & editing (equal). **Wei‐Nan Guo:** Conceptualization (equal); Writing‐review & editing (lead). **Yi‐Zhou Tan:** Investigation (equal); Writing‐original draft (supporting). **Kun‐Wei Niu:** Methodology (equal). **Jia‐Jia Zhang:** Investigation (supporting). **Cheng‐Li Liu:** Methodology (supporting). **Xiang‐Min Yang:** Methodology (supporting). **Zhi‐Nan Chen:** Supervision (equal). **Kai‐Shan Tao:** Conceptualization (supporting); Funding acquisition (equal); Supervision (lead). **Jing‐Yao Dai:** Conceptualization (lead); Data curation (lead); Funding acquisition (lead).

## Supporting information

Fig S1

Fig S2

Fig S3

Table S1

Table S2

## Data Availability

The data generated and analysed during the current study are available from the corresponding author on reasonable request.
