# Supplementary material for: Wogonin alleviates liver injury in sepsis through Nrf2‐mediated NF‐κB signalling suppression
Source: J Cell Mol Med. 2021 May 12;25(12):5782–98. doi: 10.1111/jcmm.16604 (PMC8184690; doi:10.1111/jcmm.16604)
Supplement: Supplementary file 1 — Fig S1 [file JCMM-25-5782-s005.pdf]

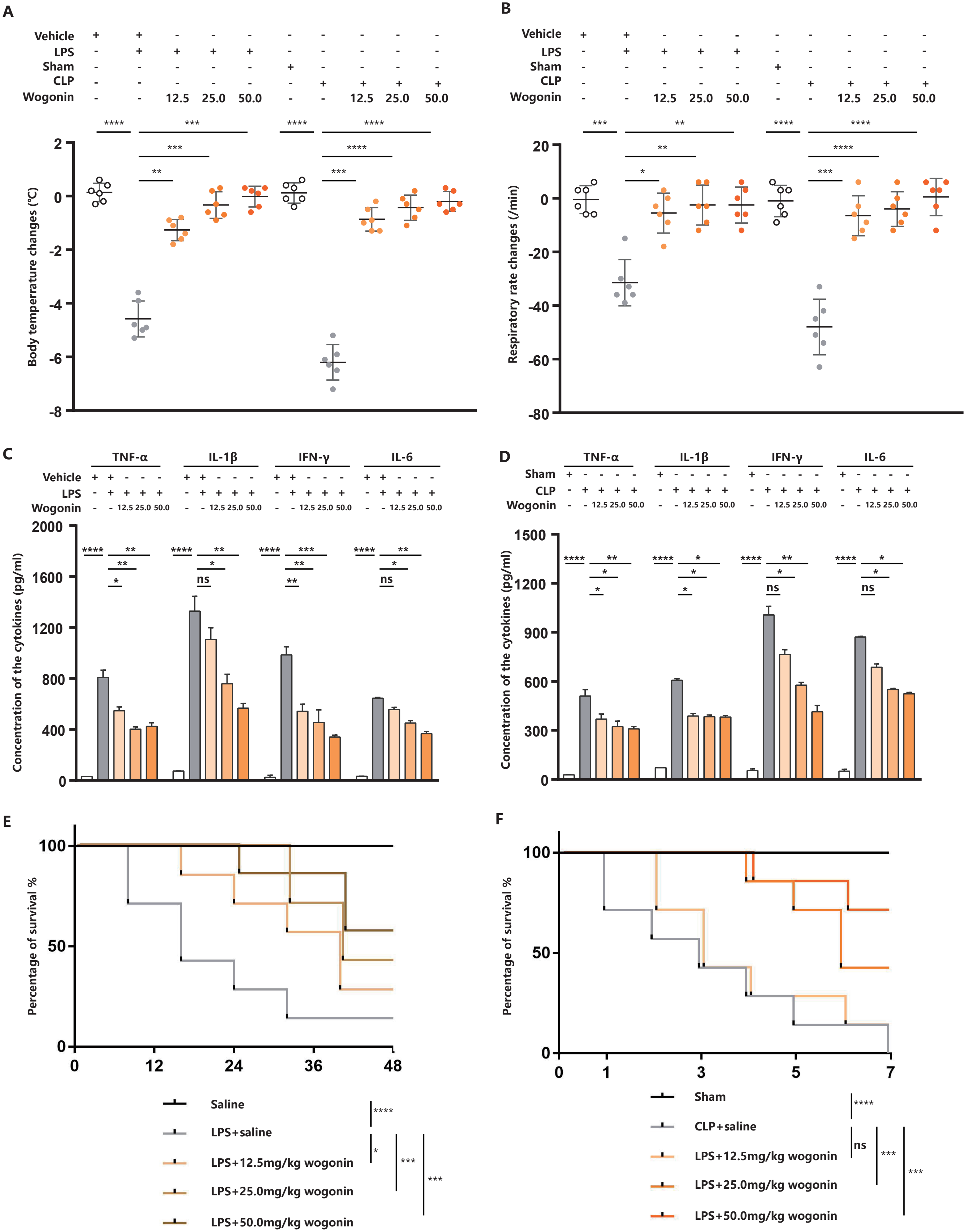

A
Vehicle
LPS
Sham
CLP
Wogonin
12.5 25.0 50.0
Body temperrature changes (℃)
B
Respiratory rate changes (/min)
C
TNF-α
IL-1β
IFN-γ
IL-6
Concentration of the cytokines (pg/ml)
D
E
Percentage of survival %
Saline
LPS+saline
LPS+12.5mg/kg wogonin
LPS+25.0mg/kg wogonin
LPS+50.0mg/kg wogonin
F
Sham
CLP+saline
LPS+12.5mg/kg wogonin
LPS+25.0mg/kg wogonin
LPS+50.0mg/kg wogonin
