# Supplementary figures and images for: Wogonin alleviates liver injury in sepsis through Nrf2‐mediated NF‐κB signalling suppression
Source: J Cell Mol Med. 2021 May 12;25(12):5782–98. doi: 10.1111/jcmm.16604 (PMC8184690; doi:10.1111/jcmm.16604)

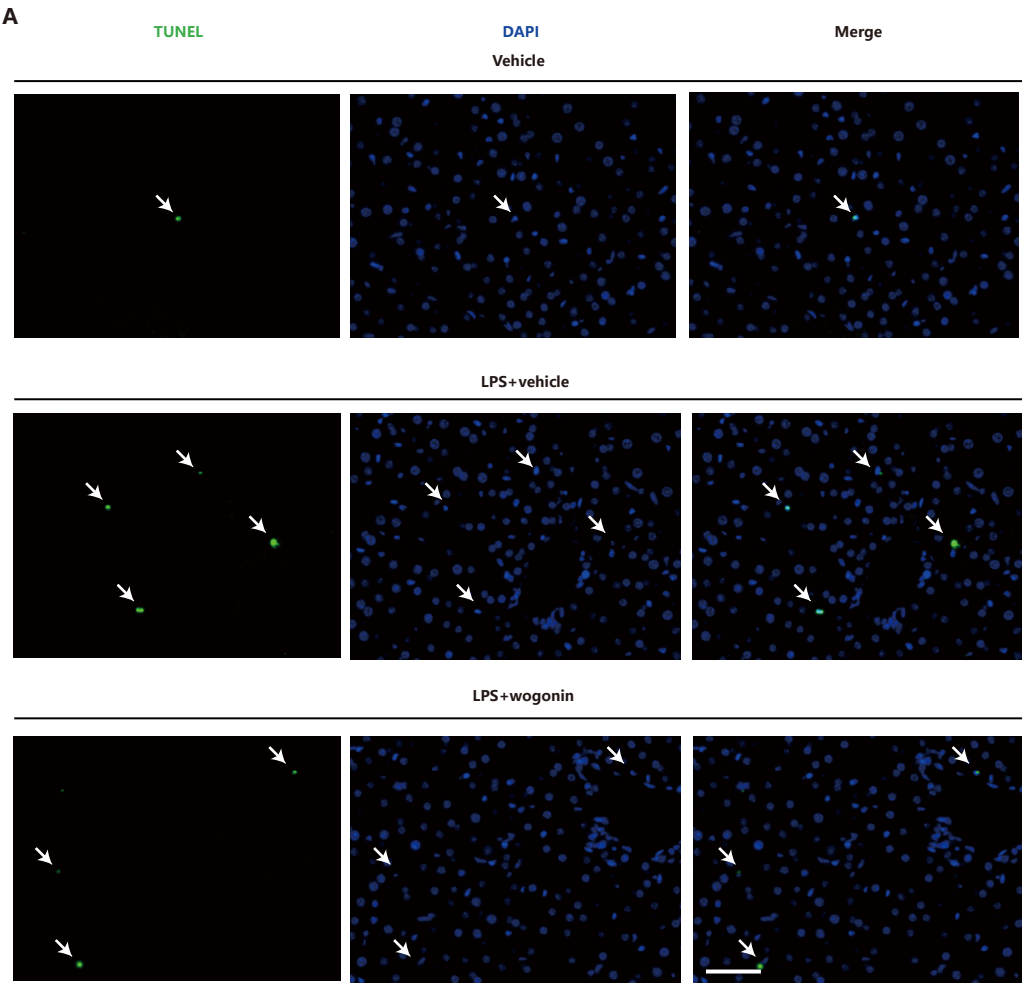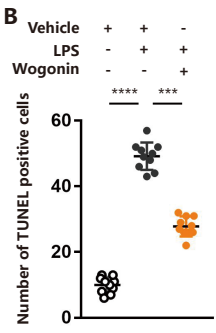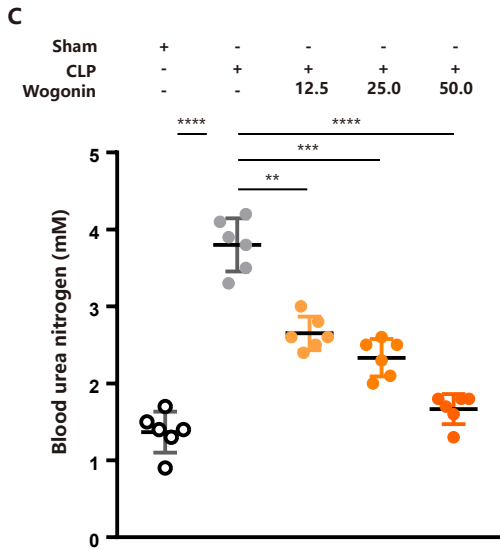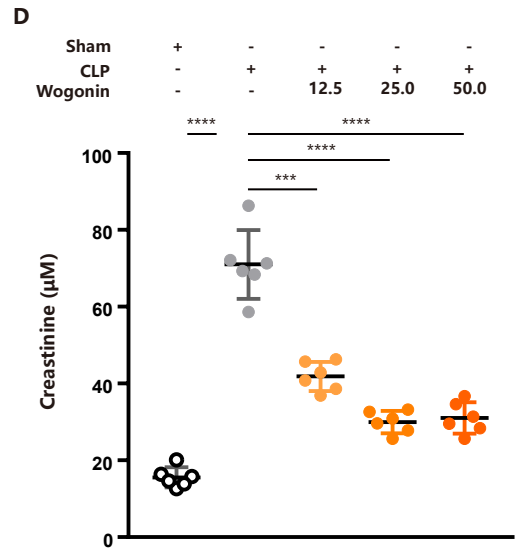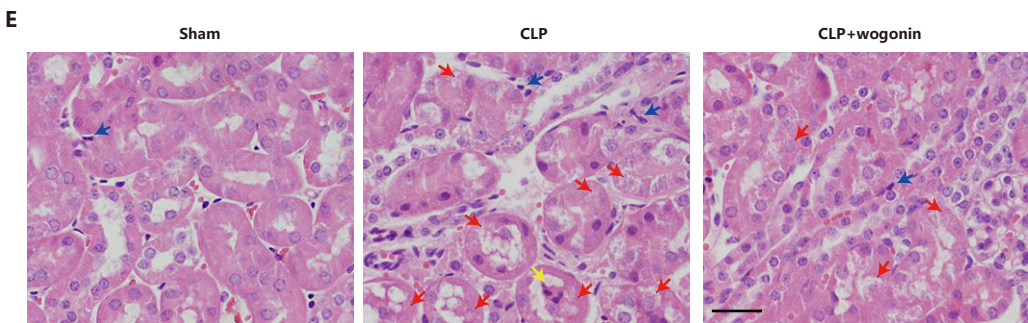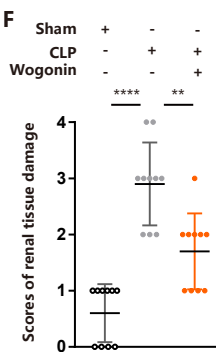

Supplement: Supplementary file 2 — Fig S2 [file JCMM-25-5782-s004.pdf]

## A Nrf2

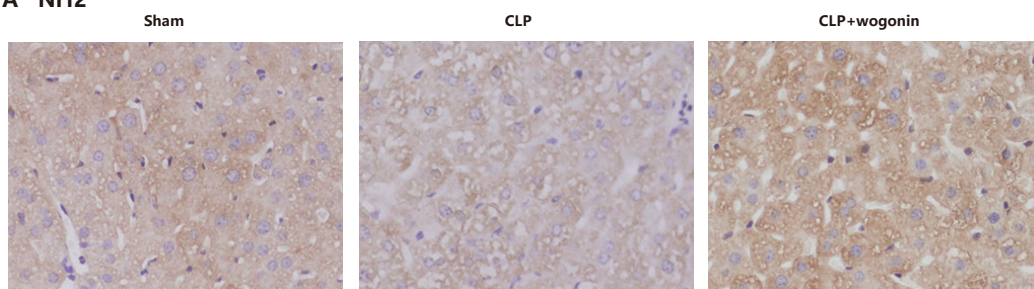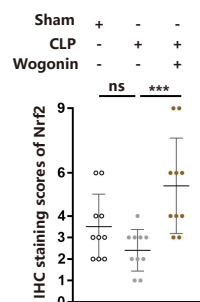

## B HO-1

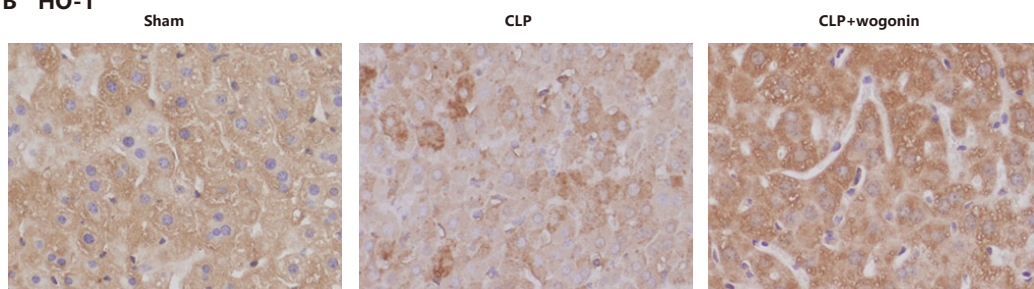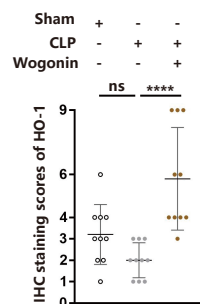

## C SOD1

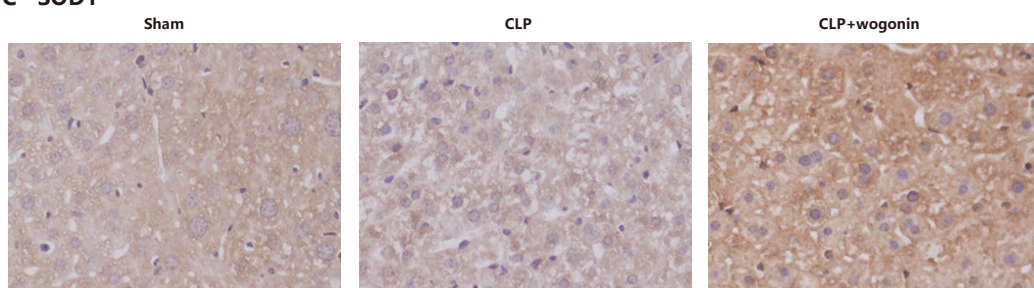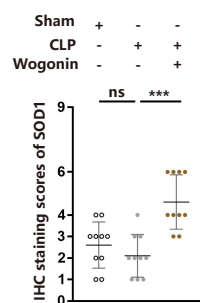

## D SOD2

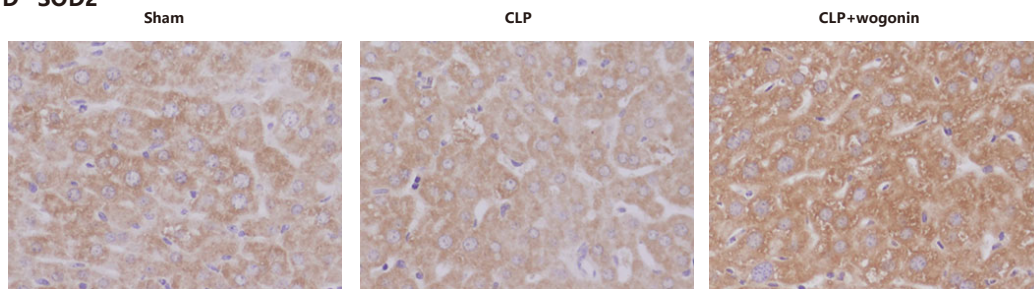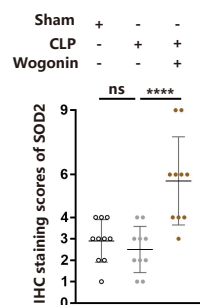

## E p-p65 (Ser536)

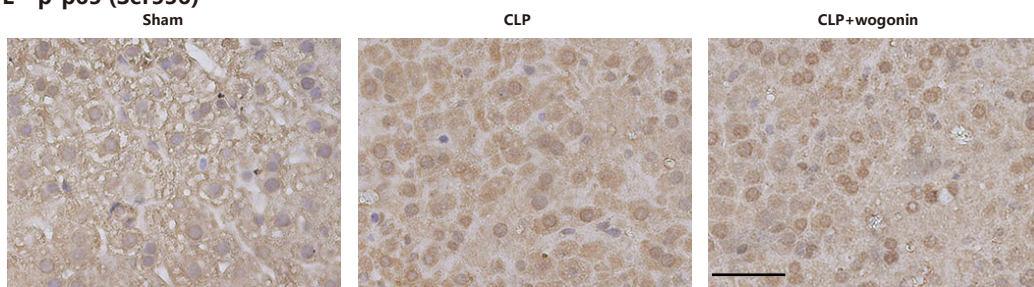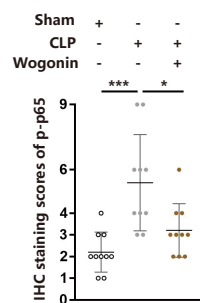

Supplement: Supplementary file 3 — Fig S3 [file JCMM-25-5782-s002.pdf]
