# Supplementary material for: Wogonin alleviates liver injury in sepsis through Nrf2‐mediated NF‐κB signalling suppression
Source: J Cell Mol Med. 2021 May 12;25(12):5782–98. doi: 10.1111/jcmm.16604 (PMC8184690; doi:10.1111/jcmm.16604)
Supplement: Supplementary file 4 — Table S1 [file JCMM-25-5782-s003.docx]

**SUPPLEMENTARY TABLE 1. Sequence information for real time PCR primers used in described studies**

| **Name** | **Primers (5′→3′)** | **Product (bp)** |
| --- | --- | --- |
| *ACTB*  *TNF*  *IL1B*  *IL6*  *NFE2L2*  *HMOX1*  *SOD1* | F^1^: GGCTGTATTCCCCTCCATCG  R^2^: CCAGTTGGTAACAATGCCATGT  F: CAGGCGGTGCCTATGTCTC  R: CGATCACCCCGAAGTTCAGTAG  F: CTGTGACTCATGGGATGATGATG  R: CGGAGCCTGTAGTGCAGTTG  F: CTGCAAGAGACTTCCATCCAG  R: AGTGGTATAGACAGGTCTGTTGG  F: TCTTGGAGTAAGTCGAGAAGTGT  R: GTTGAAACTGAGCGAAAAAGGC  F: AAGCCGAGAATGCTGAGTTCA  R: GCCGTGTAGATATGGTACAAGGA  F: AACCAGTTGTGTTGTCAGGAC  R: CCACCATGTTTCTTAGAGTGAGG | 154  89  75  131  140  100  139 |
| *SOD2* | F: CAGACCTGCCTTACGACTATGG  R: CTCGGTGGCGTTGAGATTGTT | 113 |
| *NQO1* | F: AGGATGGGAGGTACTCGAATC  R: AGGCGTCCTTCCTTATATGCTA | 144 |
| *GSTM1* | F: ATACTGGGATACTGGAACGTCC  R: AGTCAGGGTTGTAACAGAGCAT | 349 |

^1^F (forward primer), ^2^R (reverse primer)
