# Supplementary material for: Wogonin alleviates liver injury in sepsis through Nrf2‐mediated NF‐κB signalling suppression
Source: J Cell Mol Med. 2021 May 12;25(12):5782–98. doi: 10.1111/jcmm.16604 (PMC8184690; doi:10.1111/jcmm.16604)
Supplement: Supplementary file 5 — Table S2 [file JCMM-25-5782-s001.docx]

| **SUPPLEMENTARY TABLE 2. siRNA sequences used in described studies** | |
| --- | --- |
| **Name** | **siRNA sequence (5′→3′)** |
| *siNC* | F: UUCUCCGAACGUGUCACGUTT  R: ACGUGACACGUUCGGAGAATT |
| *siNrf2* | F: GCCUUACUCUCCCAGUGAATT  R: UUCACUGGGAGAGUAAGGCTT |
